# Outbreak of *Saprochaete clavata* Sepsis in Hematology Patients: Combined Use of MALDI-TOF and Sequencing Strategy to Identify and Correlate the Episodes

**DOI:** 10.3389/fmicb.2020.00084

**Published:** 2020-01-31

**Authors:** Giuliana Lo Cascio, Marcello Vincenzi, Fabio Soldani, Elena De Carolis, Laura Maccacaro, Annarita Sorrentino, Gianpaolo Nadali, Simone Cesaro, Michele Sommavilla, Valentina Niero, Laura Naso, Anna Grancini, Anna Maria Azzini, Maurizio Sanguinetti, E. Tacconelli, Giuseppe Cornaglia

**Affiliations:** ^1^Microbiology and Virology Unit, Department of Pathology, Azienda Ospedaliera Universitaria Integrata di Verona, Verona, Italy; ^2^Infectious Disease Unit, Department of Diagnostics and Public Health, University of Verona, Verona, Italy; ^3^Dipartimento di Scienze di Laboratorio e Infettivologiche, Fondazione Policlinico Universitario Agostino Gemelli IRCCS, Rome, Italy; ^4^Haematology Unit, Department of Medicine, Azienda Ospedaliera Universitaria Integrata di Verona, Verona, Italy; ^5^Division of Pediatric Oncohaematology, Department of Pediatrics, Azienda Ospedaliera Universitaria Integrata di Verona, Verona, Italy; ^6^Direzione Medica Ospedaliera, Azienda Ospedaliera Universitaria Integrata di Verona, Verona, Italy; ^7^Sezione di Igiene e Medicina Preventiva, Ambientale e Occupazionale, Dipartimento Diagnostica e Sanità Pubblica, Università di Verona, Verona, Italy; ^8^Laboratorio di Analisi Chimico – Cliniche e Microbiologia, Fondazione IRCCS Cà Granda O. Maggiore Policlinico, Milan, Italy

**Keywords:** *Saprochaete*, MALDI-TOF, yeast sepsi, outbreak, whole-genome sequencing

## Abstract

**Introduction:**

New fungal species are increasingly reported in immunocompromised patients. *Saprochaete clavata* (*S. clavata*), an ascomycetous fungus formerly called *Geotrichum clavatum*, is intrinsically resistant to echinocandins and is often misidentified.

**Objective:**

We describe a cluster of seven *S. clavata* infections in hospitalized hematology patients who developed this rare fungemia within a span of 11 months. Three of the seven patients died. Identification of the isolates was determined only with the Saramis database of VitekMS system and sequencing of the internal transcribed spacer (ITS) region. Clonal relatedness of the isolates was determined by matrix-assisted laser desorption ionization–time of flight mass spectrometry (MALDI-TOF) analysis; clonal correlation between the strains was investigated by means of phylogenetic analysis, based on single-nucleotide variants (SNPs). Clinical presentation, 1–3 β-D-glucan (BG) and galactomannan (GM) antigen results and analysis of possible sources of contamination are also described with a prospective case–control study of the outbreak.

**Results:**

MALDI-TOF MS-Vitek (bioMerieux, Marcy l’Etoile, France) failed to identify the six isolates, while SARAMIS (bioMerieux, Marcy l’Etoile, France) identified the isolates as *S. clavata*. Initially, Vitek 2 identified the strains as *Geotrichum capitatum* in two of the seven cases. Molecular identification gave 99% homology with *S. clavata*. BG was positive in three out of six patients (range 159 to >523 pg/ml), GM results were always negative. All the isolates were resistant to echinocandins (anidulafungin, micafungin, and caspofungin) and Fluconazole, but susceptible to Flucytosine and Voriconazole. One isolate showed acquired resistance to Flucytosine and Amphotericin B during treatment. Both the correlation-based dendrograms obtained by MALDI-TOF MS (Bruker Daltonics) and MS-Vitek not only clustered six of the seven bloodstream infection (BSI) isolates in the same group, but also showed their strong relatedness. Phylogenetic analysis using SNPrelate showed that the seven samples recorded during the investigation period clustered together. We observed a split between one case and the remainder with a node supported by a *z*-score of 2.3 (*p*-value = 0.021) and 16 mutations unique to each branch.

**Conclusion:**

The use of proteomics for identification and evaluation of strain clonality in outbreaks of rare pathogens is a promising alternative to laborious and time-consuming molecular methods, even if molecular whole-genome sequencing (WGS) typing will still remain the reference method for rare emergent pathogens.

## Introduction

Invasive fungal infections (IFIs) represent a public health burden worldwide, especially in immunocompromised patients, and their incidence has risen over the last decades due to the increased use of immunosuppressive and cytotoxic therapies, improved diagnostic techniques, greater awareness, and clinical suspicion. They are still a major cause of morbidity and mortality in patients with hematology malignancies (Girmenia et al., 2005; Miceli et al., 2011; Garcia-Ruiz et al., 2013; Picard et al., 2014; Stanzani et al., 2019). *Candida* and *Aspergillus* spp. play a major role. However, as a result of new prevention strategies, changes in host features and treatment protocols, new species are increasingly reported as agents of blood stream infections (BSIs) or disseminated fungi such as *Trichosporon* and *Geotrichum* (Girmenia et al., 2005; Miceli et al., 2011; Litvintseva et al., 2014; Picard et al., 2014). Particularly, *Geotrichum*, an arthroconidial yeast-like filamentous fungus whose nomenclature has recently been modified, demands special attention owing to recent outbreaks in hematology patients. Because our knowledge of these new species is poor, although improving, it is still difficult to distinguish between one fungus and another. For example, *Magnusiomyces clavatus*, formerly known as *Saprochaete clavata* or *Geotrichum clavatum*, is phylogenetically and phenotypically closely related to *Magnusiomyces capitatus*, formerly called *Geotrichum capitatum*. Both species are often misidentified and have been implicated in sporadic cases and outbreaks of fungemia mostly, but not only, in Mediterranean countries (Garcia-Ruiz et al., 2013; Desnos-Ollivier et al., 2014; Del Principe et al., 2016). Because of this microbiological flaw, and their intrinsic resistance to echinocandins and fluconazole, these infections restrict therapeutic options and are associated with high mortality rates. Here we present a prospective case–control study about an outbreak of *S. clavata* BSIs in adult and pediatric hematology patients of the Azienda Ospedaliera Universitaria Integrata di Verona, Italy, which occurred between September 2016 and July 2017. In order to provide clues about the isolates’ relatedness, typing by matrix-assisted laser desorption ionization–time of flight mass spectrometry (MALDI-TOF) analysis was performed. Finally, clonal correlation between the strains was investigated by means of phylogenetic analysis based on single-nucleotide variants (SNPs). Clinical presentation, 1–3 β-D-glucan (BG), and galactomannan (GM) antigen results and analysis of possible sources of contamination are also described.

## Materials and Methods

### Epidemiology Study

In order to limit the spread and to look for a common source of the infection, the hospital infection control and epidemiology teams, by agreement with the Medical Direction, organized a case–control study. From September 2016 to July 2017 a matched density-based sampling case–control study was undertaken, in order to investigate risk factors for *Saprochaete* fungemia in hematology patients hospitalized in the Azienda Ospedaliera Universitaria Integrata di Verona, Italy. Included cases were patients whose blood cultures proved positive for *Saprochaete* and who were hospitalized in the hematology, bone marrow transplant, and pediatric onco-hematology wards. Controls were patients hospitalized within 2 months prior to the isolation of *Saprochaete* from blood cultures of each case and with a minimum of 10 days’ hospitalization in the same wards. Each case was matched with five controls, based on hospital ward, during clinical presentation of fungal sepsis. Information about comorbidities, chemotherapy, antimicrobial therapy, presence of indwelling catheters, transfusions, and nutrition were collected by examining patient medical files, food database from the organization which provided patient meals, Hematology database, and the Hospital Pharmacy. Given this rare infection, it has not been possible to precisely estimate the sample size and the statistical power. Owing to scant literature on this topic, almost always limited to small cluster epidemics and case series, we chose to perform a conditioned regression logistic analysis, considering a *p-*value < 0.05 as statistically significant.

This retrospective observational study was based on anonymized patient data, and was carried out in accordance with the Declaration of Helsinki, under the terms of relevant local legislation, and was cleared by the Institutional Review Board. The requirement for informed consent was waived due to the observational and retrospective nature of this study.

### Microbiological Study

The Bact/Alert3D (bioMerieux, Marcy l’Etoile, France) system was used for blood cultures. A minimum of two culture vials per patient in 24 h, one fastidious antibiotic neutralization (FAN) aerobic bottle and one FAN anaerobic bottle (bioMerieux, Marcy l’Etoile, France), were filled directly with blood according to the manufacturer’s instructions. Cultures were incubated for a maximum of 5 days. On positive bottles gram staining was performed to allow direct microscopy, cultures were inoculated and incubated on Blood agar, Chocolate agar, Columbia agar (supplemented with 5% sheep blood), Sabouraud dextrose agar, and Schaedler agar under aerobic, micro-aerobic, and anaerobic conditions, respectively.

Identification of the blood isolates was undertaken using various methods, namely MS Vitek MALDI-TOF, SARAMIS, and VITEK 2(System (bioMerieux, Marcy l’Etoile, France). Primers ITS1 and ITS4 were used to amplify rDNA ITS region (White et al., 1990), while primers NL1 and NL4 were used to amplify rDNA D1–D2 region (Kurtzman and Robnett, 1997). Amplified sequences were compared with the GenBank Nucleotide Database^1^ using the algorithm Blast N (Altschul et al., 1997; de Hoog and Smith, 2004; Nilsson et al., 2006; Bader et al., 2011).

Serum BG levels were determined by the Food and Drug Administration-approved Fungitell Assay (Associates of Cape Cod, Falmouth, MA, United States) according to the manufacturer’s instructions (Budhavari, 2009). BG values < 60 pg/mL were interpreted as being negative, >80 pg/mL as being positive, and 60–79 pg/mL as indeterminate.

Serum GM levels were determined by Platelia Aspergillus (Bio-Rad, Marnes-la-Coquette, France) according to the manufacturer’s recommendations. As cleared by the US FDA the results were analyzed using an optical density (OD) index cut-off value of 0.5 on serum. The test was considered positive with an OD index value of ≥0.8 or more rather than with an OD index of 0.5 in two consecutive samples (Favre et al., 2016).

*In vitro* susceptibilities to amphotericin B, fluconazole, itraconazole, voriconazole, posaconazole, anidulafungin, caspofungin, micafungin, and flucytosine were determined by Sensititre YeastOne Y10 panel (Thermo Fisher Inc.). Readings were taken at 24 and 48 h of incubation.

In order to provide clues about the isolates’ relatedness, MALDI-TOF analysis was performed after the acquisition of the mass spectrum profiles in positive linear mode by a Microflex LT mass spectrometer (MSP, Bruker Daltonics) in accordance with the manufacturers’ recommendations. Strains of *M. capitatus* and *S. clavata* belonging to collected strain of UCSC-Rome were evaluated to show any correlation. Following MSP creation from the seven isolates, a score-oriented dendrogram was generated from hierarchical cluster analysis using the integrated statistical tool of the BioTyper 3.1 software. Hence, the same analysis was done with SARAMIS software connected to MS-Vitek in accordance with the manufacturers’ instructions. To evaluate any aspecific correlation, strains of *S. clavata* and *Geotrichum candidum* were submitted for the same analysis.

An environmental study was conducted to investigate possible sources of infection. Medical devices, drugs, blood-derived products and foods, in particular dairy products, were cultured for enrichment in Brain hearth infusion (BHI) broth and subculture in Blood agar, Chocolate agar, Columbia agar (supplemented with 5% sheep blood), and Sabouraud dextrose agar.

### Genotyping Methods

**FIGURE 1:**
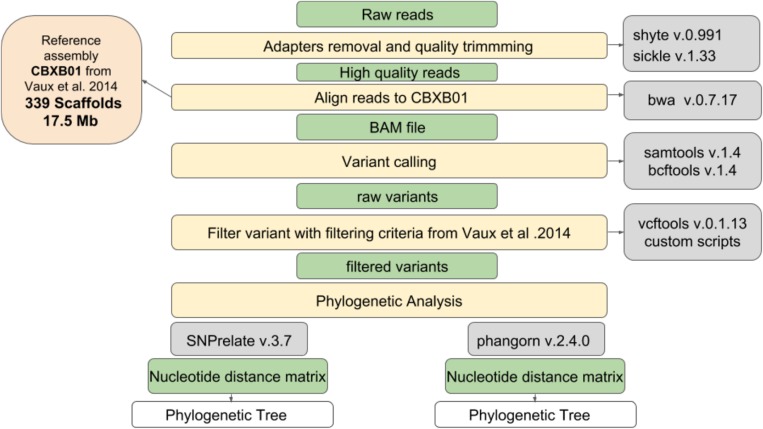
Workflow used to produce the phylogenetic tree of isolated *Saprochaete*.

All the strains of *S. clavata* occurring in the Verona outbreak and three strains of *Saprochaete capitata*, which had been isolated in another Italian hospital (Milan), were used to compare clonal correlation. All yeasts were sequenced. PCR-free DNAseq libraries were generated with the KAPA Hyper prep. Library kit. The sequencing process was based on an Illumina NextSeq500 using 2 × 150 bp-reads, analyzing 19.3 million fragments per sample on average. Figure 1 shows the workflow used in this study, which involved a number of steps from raw fastq files to the production of a phylogenetic tree. In the first step, adapters removal and quality trimming were performed using shyte v.0.991 and sickle v.1.33 software to obtain high quality reads. Then, reads were aligned to the *S. clavata* reference assembly produced by Vaux et al. (2014) (17.5 Mb, 339 scaffolds), using mem algorithm of burrows wheeler aligner v.0.7.17 with default options except when a threads argument was available. The BAM files obtained from reads alignment were used to call variants. This step in the pipeline was done using mpileup command from samtools v.1.4. The genotyping was performed using *call* command from bcftools v.1.4. All the variant calls were filtered following criteria set in Vaux et al. (2014): (i) all the variants were supported at least with four reads for each orientation; (ii) all the reads with a mapping quality above 30 were considered; (iii) variants at <100 bp from the margins of a scaffold were excluded; and (iv) the variable characters were parsimony informative (containing more than one type of nucleotide, occurring at least in two isolates) to support phylogenetic clustering. Phylogenetic correlation was inferred from high quality SNPs. R packages SNPrelate v.3.7 and Ape/Phangorn v.2.4.0 were used to compute a nucleotide distance matrix and then to perform the clustering step using a neighbor joining algorithm. To support the phylogenetic analysis with a different clustering technique, the principal component analysis was applied. Normalization of SNPs number per scaffold length was performed to check for hypervariable regions possibly connected to Fungi adaptations. The hypervariable scaffold sequences were aligned with “Basic Local Alignment Search Tool” to search for similarities with NCBI database.

## Results

**FIGURE 2:**
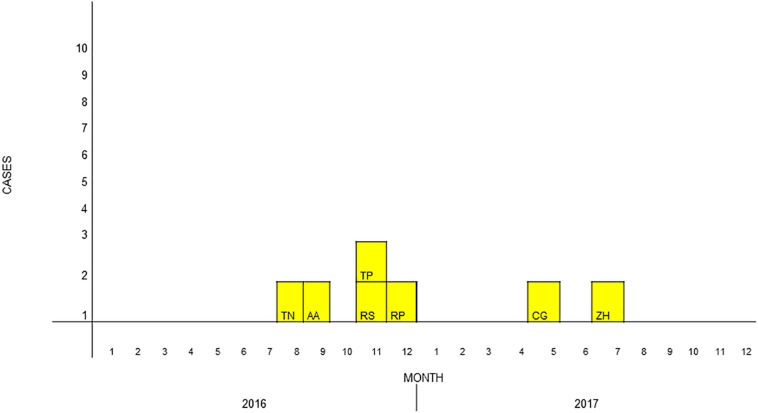
Epidemiological curve of *Saprochaete clavata* infections. The cases are designated with letters which identify the isolate, with boxes corresponding to the date of the first positive blood culture.

During the study period, a total of seven cases of *S. clavata* BSIs occurred in different hematology wards of our hospital (Figure 2). Thirty-five controls were enrolled. Table 1 summarizes the main characteristics of cases and controls. All but one of the patients were Italians. The mean ages of cases and controls were 41.1 and 51 years, respectively. Six (85.7%) cases and 18 (51.4%) controls were male. Underlying disease was acute myeloid leukemia (AML) in two of the seven cases, non-Hodgkin lymphoma in two, acute T-lymphoblastic leukemia in one, and other unspecified hematology diseases in the remaining two. It is noteworthy that all of the cases had a central venous catheter (CVC) inserted at clinical presentation of sepsis, with a mean duration of 64 days, and positive blood cultures. All of them had undergone a course of chemotherapy before the infection occurred (three out of seven with cytarabine), they were neutropenic at the time of fungemia and were also given broad-spectrum antibiotics. On univariate analysis, risk factors strongly correlated to development of fungemia were the duration of neutropenia (*p* = 0.011; OR = 1.1; 95% CI, 1.0–1.18), the receipt of plasma transfusions (*p* = 0.022; OR = 13.7; 95% CI, 1.4–128.7), the number of transfusions of blood and platelets (*p* = 0.029; OR = 1.4; 95% CI, 1.0–1.8), the use of aminoglycosides (*p* = 0.025; OR = 12.5; 95% CI, 1.4–113.2) and meropenem (*p* = 0.011; OR = 8.8; 95% CI, 1.7–47.0) (Table 2).

**TABLE 1:** Characteristics of the *Saprochaete* cases.

**Characteristics**	**Cases *N* (%)**	**Controls *N* (%)**
Sex female add a line with number of male patients and controls?	1 (14.3)	17 (48.6)
Mean age (SD)	41.1 (26.1)	51.0 (22.0)
**Age in range**		
0–40	3 (42.7)	8 (22.3)
41–50	1 (14.3)	4 (11.4)
51–60	1 (14.3)	9 (25.7)
61–70	2 (28.6)	10 (28.6)
>70	–	4 (11.4)
Transfer to other UO what is UO?	2 (28.6)	10 (28.6)
**Underlying diseases or conditions**		
Acute myeloid leukemia	2 (28.6)	10 (28.6)
Chronic myeloid leukemia	–	2 (5.7)
Lymphoma	2 (28.6)	14 (40.0)
Multiple myeloma	–	3 (8.6)
Other	3 (42.9)	6 (17.1)
Charlson co-morbidity score, media (DS)	3 (1.8)	4.3 (2.0)
**Charlson score in range**		
0–3	4 (57.1)	11 (31.4)
4–7	3 (42.9)	21 (60.0)
8–11	–	3 (8.6)
Barthel scale, mean (SD)	86.7 (21.8)	88.2 (19.6)
Dead, *N*(%)	3 (42.9)	2 (5.7)
Presence of CVC during recovery	7 (100)	32 (31.4)
**Days with CVC**		
0–20	2 (28.6)	18 (51.4)
21–40	–	10 (28.6)
>40	5 (71.4)	7 (20.0)
Days of CVC, mean (IQR)	64(18–78)	20(15–30)
Mechanical ventilation	1 (14.3)	2 (5.7)
Bladder catheter	3 (42.9)	9 (25.7)
Chemotherapy	7 (100)	29 (82.9)
Immunosuppression	7 (100)	24 (68.6)
Antibiotics	7 (100)	33 (94.3)
Aminoglycosides	6 (85.7)	11 (31.4)
Days with aminoglycosides, mean (SD)	11.4 (11.6)	0 (0.7)
Penicillin with beta-lactamases inhibitor	5 (71.4)	17 (48.6)
Days with penicillin with BL inhibitor, mean (SD)	5.5 (5.1)	4.0 (5.9)
Cephalosporins 3–4° gen.	3 (42.9)	13 (37.1)
Days with cephalosporins 3–4° gen., mean (SD)	7.1 (11.7)	3.9 (7.1)
Fluorochinolons	2 (28.2)	13 (37.1)
Days with fluorochinolons, mean (SD)	3.8 (9.4)	7.0 (11.1)
Glycopeptides, *N*(%)	4 (57.1)	9 (25.7)
Days with glycopeptides, mean(SD)	8.4 (12.0)	2.7(85.2)
Meropenem	5 (71.4)	5 (14.3)
Days with meropenem, mean (SD)	7.3 (10.0)	2.1 (8.6)
Co-trimoxazole	1 (14.3)	5 (14.3)
Days with co-trimoxazole, mean (SD)	0.3 (0.8)	0.6 (1.7)
Antifungals	4 (57.1)	26 (74.3)
Amphotericin	3	4
Days with amphotericin B, mean (SD)	2.9 (4.3)	2.6 (9.9)
Azols	2	25
Days with azols, mean (SD)	9.4 (19.5)	13.6 (11.5)
Echinocandins	2	2
Days with echinocandins, mean (SD)	3 (5.5)	0.4 (1.8)
HSCT	2 (28.6)	8 (22.9)
Days from the HSCT, mean (SD)	33 (22.6)	20.1 (9.0)
Proton pump inhibitors (PPI)	4 (57.1)	24 (68.6)
Neutropenia	7 (100)	30 (85.7)
Nadir of neutrophils, mean (DS)	11.4 (3.89)	121 (262.5)
Days with neutropenia, mean (DS)	23 (15.1)	9.9 (6.8)
**Days with neutropenia in range**		
0–10	3 (42.3)	18 (60.0)
11–20	–	10 (33.3)
>20	4 (57.1)	2 (6.7)
Growth factors	5 (71.4)	21 (60.0)
Mucositis	3 (42.9)	7 (20.0)
Diarrhea	6 (85.7)	17 (48.6)
Transfusion with RC	7 (100)	30 (85.7)
Number of red cell transfusion, mean (SD)	12.4 (9.7)	4.1 (3.3)
Plasma transfusion	4 (57.1)	4 (11.4)
Number of plasma transfusion, mean (SD)	3.1 (3.5)	0.3(1.0)
Platelet transfusion	7 (100)	26 (74.3)
Number of platelet transfusion, mean (SD)	14.4(11.7)	2.8(3.6)

**TABLE 2:** Conditioned univariate logistic regression analysis for *Saprochaete*-positive blood cultures risk factors.

**Variable**	**OR (95% CI)**	***P*-value**
Aminoglycosides use	12.5 (1.4–113.2)	0.025
Meropenem use	8.8 (1.7–47.0)	0.011
Duration of neutropenia in days	1.1 (1.0–1.18)	0.011
Plasma transfusion	13.7 (1.4–128.7)	0.022
Number of transfused platelet unity	1.4 (1.0–1.8)	0.029
Duration of aminoglycosides therapy (days)	1.2 (1.0–1.4)	0.040

**FIGURE 3:**
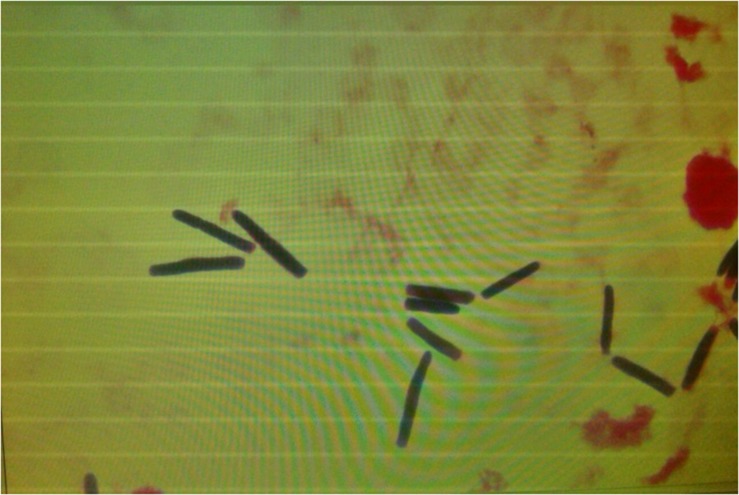
Microscopic morphology of *Saprochaete clavat*a in positive blood cultures showing yeast-like cells.

**FIGURE 4:**
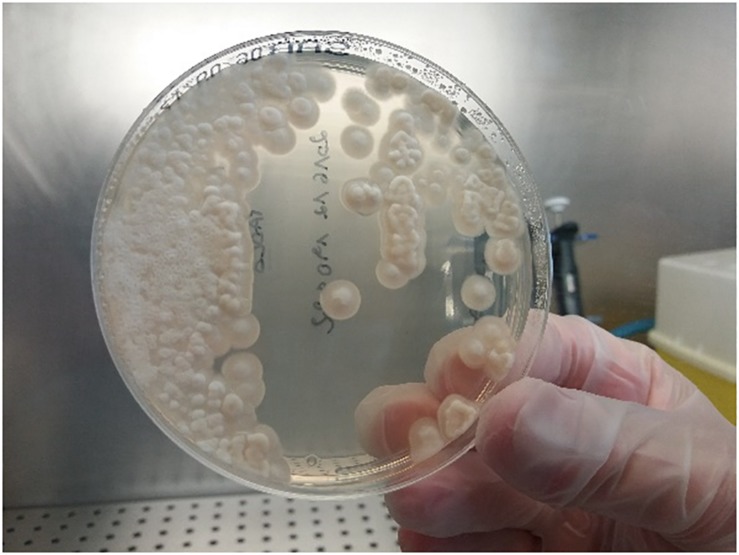
Culture of *Saprochaete clavata* on Sabouraud dextrose agar.

Microscopy of positive blood cultures showed yeast-like cells (Figure 3), while cultures on Sabouraud agar showed farinose and dry yeast colonies (Figure 4). All the isolates were from blood and one patient also had an isolate from stool. Two of the seven BSI strains were identified as *G. capitatum* by Vitek-2, while MALDI-TOF MS-Vitek (bioMerieux, Marcy l’Etoile, France) did not give any precise identification. Use of RUO SARAMIS MALDI-TOF database (bioMerieux, Marcy l’Etoile, France) and gene sequencing of the D1D2 and ITS1-5.8S-ITS2 regions identified all the isolates as *S. clavata* subspecies *clavata* with 99% homology.

β-D-glucan test was positive in three out of seven cases (range 159 to >523 pg/mL), while serum GM antigen results (Platelia Aspergillus – Bio-Rad, France) were all negative.

All the seven isolates were resistant to echinocandins and fluconazole (MIC for anidulafungin, micafungin, caspofungin 2, 8, and 8 mg/L, respectively; MIC for fluconazole 16 mg/L). The strains were susceptible to flucytosine (MIC range 0.12–0.25 mg/L), voriconazole (MIC range 0.25–0.5 mg/L), and amphotericin B (MIC range 0.5–1 mg/L). One isolate showed acquired resistance to flucytosine and amphotericin B during treatment.

**FIGURE 5:**
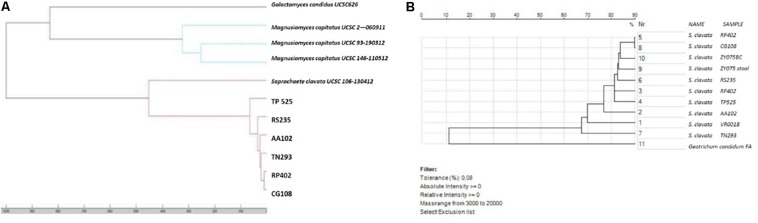
Correlation-based dendrograms obtained by **(A)** MALDI-TOF MS (Bruker Daltonics) and **(B)** MS-Vitek Saramis (bioMerieux, Marcy l’Etoile, France).

In the genotyping analysis, both the correlation-based dendrograms obtained by MALDI-TOF MS (Bruker Daltonics) and MS-Vitek not only clustered six of the seven BSI isolates in the same group, but also showed their strong relatedness (Figure 5). Bruker software correctly distinguished *M. capitatus* from *S. clavata*, and clustered the first six strains of our outbreak together. The second analysis with SARAMIS-MS Vitek software was carried out after the seventh case occurred. In the last patient (ZY075) *S. clavata* was isolated from blood and from stool and both isolates were analyzed. The SARAMIS analysis clustered these *S. clavata* strains together, except for the TN293 strain which appeared moderately far away from the others, and closest to the VR00018 strain, not involved in the outbreak. SARAMIS analysis well differentiated *S. clavata* from *G. candidum*.

The genotyping analysis was performed on *S. clavata* strains belonging to the Verona outbreak and from another hospital in Milan, during the same year.

After comparing our isolates with those from Milan, the mapping procedure gave 17.5 million mapped fragments on average, with a median coverage of 262×. The details per sample of the sequenced and mapped fragments are reported in Table 3.

**TABLE 3:** Details of sequenced fragments for each strain of *Saprochaete* isolated from Verona and Milan.

	**Sequenced**	**Mapped**	**Average**
**Sample**	**fragments**	**fragments**	**coverage**
RS235	16,282,394	14,335,384	216.59
RP402	24,467,857	22,739,883	331.26
AA102	16,692,742	14,796,911	218.73
CG108	13,899,852	12,314,272	187.31
TP525	14,058,887	12,582,330	186.30
TN293	24,179,648	18,491,452	278.89
ZY075	22,790,265	21,525,722	330.71
MI409	19,684,314	18,707,031	287.14
MI691	19,061,001	18,077,681	280.81
MI031	21,889,597	20,360,099	311.55

**FIGURE 6:**
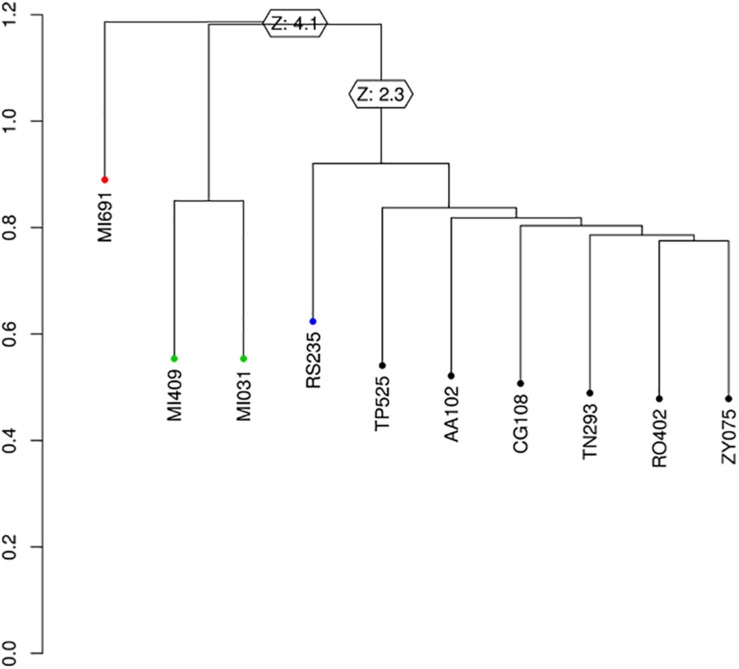
Phylogenetic tree produced by SNprelate, *z*-score at supported nodes.

**FIGURE 7:**
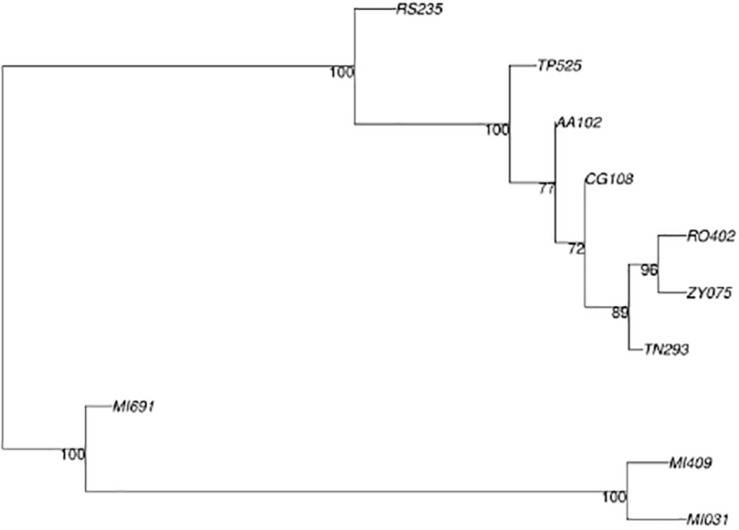
Phylogenetic tree produced by Ape/Phangorn, bootstrap value at nodes.

The variant calling procedure produced a list of 1,529 variant sites that was reduced to 246 by filtering for SNVs. The set of 246 variant sites was then used to infer phylogeny. Phylogenetic analysis using SNPrelate (Figure 6) shows that of the 10 samples recorded during the investigation period, those from Milan MI409 MI691 and MI031 belonged to one clade while the samples from Verona (RS235, TP525, AA102, CG108, TN293, RO402, ZY075) accounted for another cluster, with a node supported by a *z*-score of 4.1 (*p*-value = 0.000041), with a final difference of 61 mutations between the two clusters. In particular, inside the second cluster, we observed a split between RS235 and the rest of the samples with a node supported by a *z*-score of 2.3 (*p*-value = 0.021) and 16 mutations unique to each branch. The phylogenetic analysis using Ape/Phangorn (Figure 7) shows the same tree topology as in Figure 6.

**FIGURE 8:**
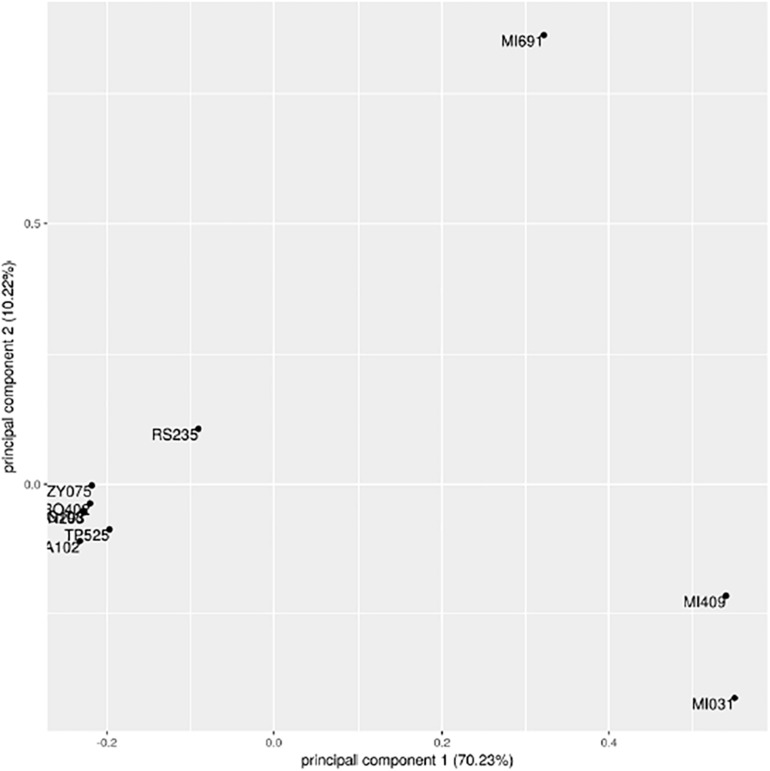
Principal component analysis of 199 informative SNPs.

Such analysis provides a bootstrap value as a measure of statistical confidence: the higher the value, the larger the number of mutations supporting the branch. In this analysis, the same nodes in Figure 7 are supported with a bootstrap value of 100 indicating full separation between the groups identified in the first analysis. The results for the first two principal components of the data set that explain together ∼80% of variation are shown in Figure 8.

The first component clearly divides the three samples MI409, MI691, and MI031 from the others. The sample RS235 is partially separated from the two main groups, consistent with results of our phylogenetic trees. Four contigs out of the 339 present in the genome assembly were identified as hypervariable; however, after alignment with blastn, none of them had significant similarities with sequences present in NCBI. Data set are available on request.

Environmental studies for possible sources of infection included microbiology investigation of different dairy products, and many medical devices and antibiotic formulations. In detail, microbiological investigation was conducted on 10 different yogurt products, 3 replicates of 3 different cheeses likely to have been eaten by patients in hospital meals, as suspected during the epidemiological investigation. Additionally, medical devices, such as vascular catheters and platelet infusion kits, were cultured. Also cultured were apheresis platelet concentrates and plasma derivatives. Two different batches of amikacin, gentamicin, and meropenem were cultured after enrichment in BHI. The results of investigations of fungal contamination of food, medical devices, blood derivatives, and antibiotics were always negative.

### Clinical Cases

Of the seven cases which occurred in Verona, clinical information are available for only four.

The first case was a 20-year-old Italian girl with a T-ALL diagnosis, admitted to our hematology ward for follow-up chemotherapy. A few days after the first infusion she became febrile with chills and broad spectrum antibiotics were given. Blood cultures turned positive for yeast-like fungi, so Caspofungin was added to therapy (Arendrup et al., 2014). The patient developed progressive arterial hypotension and was admitted to the ICU ward where she died of septic shock. The fungus, which was first isolated from blood cultures, was then identified as *G. clavatum* by means of Vitek-2. Drug susceptibility test showed a high-level resistance to fluconazole and all echinocandins.

The second case was a 65–70-year-old Italian patient with a cytotoxic NHL who was hospitalized in October 2016 in the hematology ward for follow-up chemotherapy. Before completing the cycle, he presented a high-grade fever, nausea with vomiting, and diarrhea. As well as antibiotics for CVC bacteremia due to *Pseudomonas aeruginosa*, he was given liposomal amphotericin-B for *G. clavatum* fungemia for a total of 7 days, then switched to voriconazole for a further 10 days on the basis of a drug susceptibility test. On CT scan, no disseminated disease was reported and the patient was finally discharged home.

The third case was a 4-year-old Italian child with AML, which was diagnosed when he was 1 year old. He started chemotherapy soon after diagnosis with good initial response. In October 2016 he was hospitalized because of a high-grade fever, anemia, low platelets, and leukocytosis. On blood examination, a recrudescence of his disease was evident, so new chemotherapy was infused. After a few doses, he presented with a high-grade fever, cough, and diarrhea. Broad spectrum antibiotics and antifungals were given, after collection of blood cultures (Camus et al., 2014). *G. clavatum* and *St. epidermidis* were isolated, so targeted therapy with vancomycin and desoxycholate amphotericin B was used for up to 15 days. He was then discharged home and is still under hematology follow-up.

The fourth case was a 50–60-year-old Italian patient, admitted in November 2016 to the hematology ward for relapsing AML. During hospitalization, thanks to a good HLA match, he underwent hematopoietic stem cell transplant (HSCT) after induction chemotherapy. Five days after HSCT, he began to complain of fever with chills, vomiting, and diarrhea. Despite being on antifungal prophylaxis with Posaconazole, he developed *S. clavata* fungemia. On PET-CT scan no disseminated disease was reported. He received liposomal amphotericin B for 21 days with no relapse and was discharged home 1 month later.

The three cases in Milan were comparable for age and clinical history. They were Caucasian adult patients, admitted to the hematology ward to start chemotherapy for AML. Because of fever, blood was drawn for cultures. After being misidentified as *S. clavata*, the strains were then recognized as *S. capitata*. AMB-L and 5-FC therapy was started with resolution of symptoms.

## Discussion

While the general population is aging and experiencing multiple chronic comorbidities, together with new and increasingly complex therapies which strongly impact immune status, clinical suspicion of infectious diseases caused by rare pathogens must arise. Among these, fungi are playing an important role because they are difficult-to-treat, slow progressing infections with high morbidity and mortality. Unlike *Aspergillus* and *Candida*, little is known about *Saprochaete* spp., formerly known as *Geotrichum*, in immunosuppressed patients in terms of epidemiology, source of outbreaks, risk factors, treatment, and outcomes.

The taxonomic classification of *Geotrichum* is currently changing. It was originally known as *Trichosporon* spp. and classified among the basidiomycetes (Gurgui et al., 2011). Subsequently, due to its cell wall structure, septal pores, and its tendency to produce numerous arthroconidia and few blastoconidia, from a microbiological point of view it was considered to be an ascomycete (Kurtzman and Robnett, 1997). Further reclassification has been made recently, thanks to novel and finer microbiological techniques which rely on proteome and genome sequencing. *Geotrichum* species is now defined as *Saprochaete* even though some experts name it *Magnusiomyces* (Desnos-Ollivier et al., 2014; Vaux et al., 2014). Misidentification is frequent due to their similar phenotypes and limited information collected in the databases. As a consequence, differentiation may be very difficult when based on macroscopic and microscopic analysis. Colonies present white, farinose, dry with frosted glass appearance, and microscopic features consist of true hyphae organized in acute branches often disarticulated into arthroconidia with rectangular or rounded ends (Desnos-Ollivier et al., 2014).

The first case of *Geotrichum* infection was reported in 1965, and to date other cases or small outbreaks have been described, predominantly in Italy and France (Gadea et al., 2004; Girmenia et al., 2005; Del Principe et al., 2016; Esposto et al., 2018; Stanzani et al., 2019). Clinical presentations were sepsis, pulmonary hepatic and splenic lesions, brain abscesses, and were characterized by a high mortality rate, due to peculiar antifungal resistance patterns, difficult identification and, consequently, delay in diagnosis and correct treatment (Gadea et al., 2004; Cuenca-Estrella et al., 2011; Esposto et al., 2018). A 20-year observational multicenter Italian study reported 35 cases of *G. capitatum* infection, with a mortality rate of 57.1% (Girmenia et al., 2005; Esposto et al., 2018). Most of those cases occurred in patients with hematology malignancies, especially during profound neutropenia due to chemotherapy.

The optimal therapy has yet to be established and remains a challenge (White et al., 1990; Kurtzman and Robnett, 1997; Arendrup et al., 2014), because they are rare, subtle, and difficult-to-diagnose infections, with no current available breakpoints from EUCAST nor CLSI and intrinsic resistance to echinocandins and fluconazole.

Here we report a small cluster of seven *S. clavata* infections occurring between September 2016 and July 2017 in the Azienda Ospedaliera Universitaria Integrata di Verona, Italy. All infections developed in adult and pediatric hematology patients. Each case was matched with five controls on the basis of the ward of hospitalization during fungal sepsis. Of note, all clinical cases had experienced severe neutropenia following chemotherapy; had a CVC or other central vascular device; were given broad-spectrum antibiotics and transfusions; and had high-grade fever with chills, diarrhea, and vomiting. Significant associations with transfusions and antibiotics may be explained by the fact that almost all cases were septic and needed stronger supportive therapies than the controls. As seen in other reports, all of our patients who had experienced *S. clavata* fungemia were severely immunocompromised, underscoring that the most important epidemiological risk factor for this kind of infection is immunosuppression and in particular neutropenia. It is noteworthy that, among patients of our hospital whose blood cultures turned positive for *S. clavata*, one tested positive also for stool, strengthening the hypothesis that this fungus can colonize the gut mucosa and migrate into the circulation or vice versa, as *Candida* spp. does. Probably, gut colonization and translocation, caused by chemotherapy-induced intestinal tract damage or mucositis, may have played an important role (Camera et al., 2003; Lee et al., 2014). In addition, we cannot exclude that *Saprochaete* could colonize the skin from the environment and invade the blood system through portals of entry such as catheters or wounds. As a retrospective study, the gut colonization of all the patients was not investigated, except for the last one.

Unlike previous historical outbreaks, mortality was not very high, thanks to rapid molecular identification and start of amphotericin B formulations, which is the recommended therapy for now, given intrinsic resistance to echinocandins and fluconazole.

*Saprochaete clavata* grew from all blood cultures and identification was made with SARAMIS-MS Vitek (bioMerieux, Marcy l’Etoile, France) and nucleotide sequencing. Actually, whole-genome sequence (WGS) typing is the best available molecular method to evaluate the clonality of a suspected outbreak caused by uncommon fungal species (Alanio et al., 2017; Bougnoux et al., 2018). It is based on the analysis of whole single-nucleotide polymorphisms (SNPs) within each genome, which enables comparison between strains. The number of different SNPs and their distances allow for inference of strains relatedness. Phylogenetic analysis using SNPrelate (Figure 6) showed that the seven samples from the Verona outbreak belonged to one clade. We observed a split between RS235 and the rest of the samples caused by 16 mutations, but further and more robust studies are needed to explain their significance. Vaux et al. (2014) described a nationwide outbreak which occurred in the spring of 2012 involving 10 health-care facilities in France, due to one clade of *S. clavata.* Even though they made a nationwide microbiological and epidemiological investigation, they did not find the source of infection; however, food, especially dairy products, or apheresis platelet concentrates were strongly suspected. In our outbreak we observed seven cases in a very short period of time and we deemed that a common, easily diffusible source of infection could have been present in our hospital. We checked for diverse potential vehicles of infection although microbiology results were always negative. Because, during neutropenia and chemotherapy-induced mucositis the risk of gut translocation is high, we thought that food could have been one of the most likely sources. We then tested and cultured some dairy products (yogurt, creamy cheese), as our French colleagues had done before. Unfortunately, the retrospective nature of food investigation precluded collection of all potential sources of contamination, and microbiology gave negative results. Aside from foods, medical devices, and drugs, we did not perform culture investigation on healthcare providers as potential vectors of infection. As such, we cannot make conclusions on how the epidemic began; microbiology was always negative, so we could neither find a source of infection nor implement a plan to control or avoid it.

This is the largest outbreak by a rare fungus ever observed in our hospital, worthy to be described, and evaluated from a microbiological point of view. One strength of our work is that the clonal relatedness of our isolates relied on a proteomic approach, using two of the most diffuse mass spectrometry machines available in microbiology clinical laboratories, the Bruker and the Biomerieux MS Vitek. The clonality of the strains was evidenced by the clustering during proteomic evaluation. Both Bruker MALDI Biotyper system and bioMerieux MS-VITEK showed high similarity of the isolates. Interestingly, both systems highlighted that one different isolate did not strictly correlate with the other six. Few other reports have tried to discuss the usefulness of proteomic fungal typing by means of MALDI-TOF, which was able to describe and confirm strains’ relatedness in an outbreak (Stanzani et al., 2019).

To date many papers have shown the ability of MALDI-TOF in accurately distinguishing different clusters of *Candida* strains, geographically grouped as in the case of *Candida auris* (Pracash et al., 2016) or in the case of multidrug resistant *Candida glabrata* (Dhieb et al., 2015) and *Candida parapsilosis* complex (De Carolis et al., 2014). MALDI-TOF mass spectra for genotyping would represent a rapid, first-level, easy, and cost-effective way to support clinicians, during uncommon fungal outbreaks, in treatment decision-making and in a clinical microbiology lab, although only WGS can confirm it. Of course, further studies are needed to strengthen the accuracy and efficacy of MALDI-TOF as a fungal typing tool even in rare infections. Although rare, *S. clavata* should be considered as a potential cause of IFI, particularly in cases of severe neutropenia. Last, but not least, its intrinsic resistance to echinocandins and fluconazole must be considered when selecting an empirical antifungal therapy as this infection may be rapidly lethal. National surveillance is desirable to successfully investigate multicenter outbreaks of infection due to rare pathogens.

## Data Availability Statement

The raw data supporting the conclusions of this article will be made available by the authors, without undue reservation, to any qualified researcher.

## Ethics Statement

The studies involving human participants were reviewed and approved by the Institutional Review Board of Azienda Ospedaliera Universitaria Integrata di Verona. The requirement for written informed consent was waived due to the observational, retrospective nature of this study. The patients/participants provided their written informed consent to participate in this study.

## Author Contributions

GL, MS, ET, and GC contributed to the conception and design of the study. ED, LM, and AS performed the MALDI-TOF analysis. GN, SC, MS, AG, VN, and AA performed the microbiological and clinical analysis. LN performed the molecular analysis. MV and FS performed the case-control study.

## Conflict of Interest

The authors declare that the research was conducted in the absence of any commercial or financial relationships that could be construed as a potential conflict of interest.

## Footnotes

1 http://www.ncbi.nlm.nih.gov
